# Exposure to a fungal pathogen increases the critical thermal minimum of two frog species

**DOI:** 10.1002/ece3.7779

**Published:** 2021-06-24

**Authors:** Spencer R. Siddons, Catherine L. Searle

**Affiliations:** ^1^ Department of Biological Sciences Purdue University West Lafayette IN USA

**Keywords:** amphibians, *Batrachochytrium dendrobatidis*, chytrid, chytridiomycosis, Hyla versicolor, Lithobates palustris

## Abstract

The ability of an organism to tolerate seasonal temperature changes, such as extremely cold temperatures during the winter, can be influenced by their pathogens. We tested how exposure to a virulent fungal pathogen, *Batrachochytrium dendrobatidis* *(Bd)*, affected the critical thermal minimum (CT_min_) of two frog species, *Hyla versicolor* (gray treefrog) and *Lithobates palustris* (pickerel frog). The CT_min_ is the minimum thermal performance point of an organism, which we estimated via righting response trials. For both frog species, we compared the righting response of *Bd*‐exposed and *Bd*‐unexposed individuals in either a constant (15ºC) environment or with decreasing temperatures (−1°C/2.5 min) starting from 15°C. The CT_min_ for both species was higher for *Bd*‐exposed frogs than unexposed frogs, and the CT_min_ of *H*. *versicolor* was higher than *L*. *palustris*. We also found that *Bd*‐exposed frogs of both species righted themselves significantly fewer times in both decreasing and constant temperature trials. Our findings show that pathogen exposure can reduce cold tolerance and limit the thermal performance range of hosts, which may lead to increased overwintering mortality.

## Graphical abstract

We tested how exposure to a virulent fungal pathogen, *Batrachochytrium dendrobatidis*, affected the critical thermal minimum of two frog species. In both frog species, exposure to the fungal pathogen increased the critical thermal minimum. Our findings show that pathogen exposure can reduce cold tolerance and limit the thermal performance range of hosts.

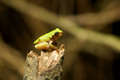

## 1 INTRODUCTION

Seasonal variation in temperature is a major force on host–pathogen interactions (Altizer et al., 2006). In temperate regions, winter is often considered a time when many host–pathogen dynamics slow or stop altogether, but many pathogens remain active (Dawson et al., 2007; Hosseini et al., 2004). Due to energetic costs of pathogen infection, disease may impede a host's physical functions necessary for winter survival, such as fall migrations or cold tolerance (Cunjak, 1986). Thus, an organism's thermal tolerance limits, the temperature ranges where an organism can maintain normal locomotor function, can be affected by pathogens (Greenspan et al., 2017; Hayman et al., 2016). For many organisms, compressed thermal tolerance limits (both lower and upper limits) can be lethal if they are unable to cool or heat as needed (Lutterschmidt & Hutchison, 1997).

Thermal tolerance limits are commonly measured to identify the thermal requirements of an individual to perform a particular function (Sunday et al., 2011). The critical thermal maximum (CT_max_) and critical thermal minimum (CT_min_) are the highest and lowest temperatures that an individual can function, respectively (Catenazzi et al., 2011; Hector et al., 2019; Lutterschmidt & Hutchison, 1997; Sunday et al., 2011). Previous work has investigated the effects of pathogens on host CT_max_ (Taylor et al., 2020). For organisms that thermoregulate to avoid thermal limits, the CT_max_ is often a cutoff for when mortality is observed, while the CT_min_ does not typically cause death, but is an endpoint for the ability to perform a given function (Layne & Roman, 1985). However, the upper thermal tolerance limits garner much of the work on host thermal limits, while relatively little is known regarding how pathogens affect host CT_min_. The potential change in CT_min_ due to pathogen infection is important to understand when evaluating performance and survival of hosts in winter.

Many hosts experience extreme physiological challenges in colder seasons that increase their susceptibility to disease, potentially intensifying the negative effects of infection in winter. For example, hosts may experience suppressed immune functions due to limited resources and altered weather conditions, such as decreasing temperatures (Dowell, 2001). Simultaneously, pathogens typically have a wide thermal performance breadth (Martiny et al., 2006), which can make them more tolerant to colder conditions than their hosts. Altered host CT_min_ of individuals in winter could have drastic effects on population viability, which may go unnoticed due to cryptic overwintering habits of many species, and the lack of disease sampling in winter.

In recent decades, multiple fungal pathogens with substantial tolerance or preference for cold conditions have been documented in temperate regions (e.g., white‐nose syndrome, snake fungal disease; Blehert et al., 2009; Allender et al., 2015). One such pathogen is the chytrid fungus, *Batrachochytrium dendrobatidis* (*Bd*), the causative agent of chytridiomycosis, which has been responsible for ongoing global amphibian declines and extinctions (Lips, 2016; Longcore et al., 1999). Infection with *Bd* occurs in the epidermis of frogs, disrupting osmoregulation, damaging tissue, and causing metabolic dysregulation (Grogan et al., 2018; Voyles et al., 2009). The optimal temperature range for *Bd* maturation and reproduction in vitro is between 17 and 25°C, but this pathogen can reproduce and transmit in water at temperatures as low as 4–5°C (Piotrowski et al., 2004; Voyles et al., 2017). Because optimal *Bd* growth occurs in temperatures that are cooler than many temperate regions in the summer, *Bd* infection prevalence and burdens are often highest in cooler months (Fernández‐Beaskoetxea et al., 2015; Longcore et al., 2007; Siddons et al., 2020), can increase through the winter (Kinney et al., 2011), and can increase mortality risk in overwintering juvenile frogs (Rumschlag & Boone, 2018). Mounting an immune response to *Bd* exposure in the form of resistance can be costly to growth, development, and survival, and alter corticosterone levels for amphibian hosts (Luquet et al., 2012; Murone et al., 2016; Savage et al., 2016). Therefore, the cost of *Bd* resistance on energetic stores can limit other physiological activities. Because it is unlikely that *Bd* infection dynamics cease completely during the winter, a time when hosts are highly susceptible to *Bd*, it is necessary to identify host responses to *Bd* exposure in cold conditions (Rachowicz & Briggs, 2007; Zapata et al., 1992).

The upper thermal limits of amphibians can be altered by *Bd*, but little is known about how *Bd* affects lower thermal limits. Frogs infected with *Bd* can experience a reduction in CT_max_, likely due to the effects of chytridiomycosis, such as inhibition of cutaneous processes and metabolic dysregulation (Fernández‐Loras et al., 2019; Greenspan et al., 2017; Grogan, Skerratt, et al., 2018). The subsequent effects of an altered CT_max_ can reduce fitness of individuals and alter population transmission dynamics if hosts congregate within a more narrow microclimate to maintain homeostasis (Duarte et al., 2012; Greenspan et al., 2017). The understanding of *Bd*‐induced changes to thermal tolerances in amphibians focuses on CT_max_, likely because this threshold generally results in rapid mortality (Taylor et al., 2020). However, it is vital to identify the impact of *Bd* on CT_min_ of amphibians that experience cold or near‐freezing temperatures. Colder conditions reduce amphibian immunocompetence and energetic stores necessary to combat *Bd* and survive winter (Auer et al., 2015; Podhajský & Gvoždík, 2016; Zapata et al., 1992). Concurrently, *Bd* matures and reproduces best in relatively cool temperatures in temperate regions, making winter a potentially high‐risk season for *Bd* outbreaks.

We explored the lower range of thermal tolerance of amphibians exposed to *Bd* to better understand how *Bd* affects its hosts in the winter. We tested if *Bd* exposure would limit the righting response of two frog species in cold temperatures. We predicted that *Bd*‐exposed individuals would have a higher CT_min_ than unexposed individuals for both species because the pathogen would reduce overall physiological function. This investigation could highlight a mechanism of *Bd* pathology and winter mortality of host species in temperate regions.

## 2 MATERIALS AND METHODS

### 2.1 Animal collection and husbandry

We collected one egg mass of *Lithobates palustris* (pickerel frog) in April 2018 and *Hyla versicolor* (gray treefrog) in May 2018 in Tippecanoe County, IN, USA. These species differ in their overwintering habitats. *Lithobates palustris* remain in lakes or streams, or migrate to caves to avoid freezing (Fenolio et al., 2005; Resetarits, 1986). *H*. *versicolor* migrate to the forest floor and tolerate freezing by distributing cryoprotectant metabolites (e.g., glycerol) to their cells to prevent intracellular ice formation (Storey & Storey, 1985). Animals were housed in 37.8 L tanks through metamorphosis. Tadpoles were fed a mixture of fish flakes, rabbit chow, and algae pellets. Postmetamorphic individuals (i.e., “metamorphs”) were fed wingless fruit flies (*Drosophila melanogaster*). Lighting matched outdoor conditions through a window until *Bd* exposure (see below).

### 2.2 Pathogen culturing and exposure

We exposed approximately half of the metamorphs from each species to *Bd*. A total of 18 *L*. *palustris* and 15 *H*. *versicolor* were exposed to *Bd*, while 18 and 14 were left unexposed, respectively. We used a *Bd* strain isolated from an infected *Lithobates sp*. from Ohio (JSOH‐1), grown on 1% tryptone agar plates for seven days and quantified using a hemocytometer.

Immediately prior to *Bd* exposure, we measured weight (g) and snout‐vent‐length (mm) for each animal. Each *Bd*‐exposed individual was then exposed to 340,000 zoospores for 24 hr in a 9‐cm (diameter) plastic petri dish with air holes and 10 ml of inoculated water (Searle et al., 2011). Unexposed individuals were given 10 ml of sham inoculated water. To ensure animals were exposed to *Bd*, petri dishes were manually tilted 12 times over the 24‐hr exposure period to allow the inoculated water to contact each individual. After the exposure period, animals were immediately placed in plastic deli‐cups lined with an un‐bleached cotton cloth saturated in water. Deli‐cups were placed into an incubator at 21°C without light, and temperature was reduced by approximately 0.3°C per day over 21 days to reach 15°C (Irwin & Lee, 2003). Frogs were fed wingless fruit flies (*D. melanogaster*) ad libitum until temperatures reached 18°C. The goal of the temperature and feeding reduction was to simulate a decrease in temperature and light that occurs during the fall leading up to winter. This change in ambient temperature and light coincides with a reduction in activity and feeding for both species near 15°C (John‐Adler et al., 1988; Resetarits, 1986). Righting response trials began once the temperature reached 15°C.

### 2.3 Righting response trials

We measured the critical thermal minimum (CT_min_) of all individuals, which is the temperature at which an individual loses locomotor function (Lutterschmidt & Hutchison, 1997). Due to mortality leading up to the trials, only 11 unexposed and seven exposed *H*. *versicolor* and 12 unexposed and six exposed *L*. *palustris*, individuals were tested. We conducted two trials under minimal light over the course of seven days. In the “constant” trial, the temperature remained at 15°C, while in the “decreasing” trial, the temperature was reduced at a rate of −1°C/2.5 min starting at 15°C. Comparing the constant temperature trial with the decreasing trial allowed us to confirm that the number of righting responses in the decreasing temperature trial was not driven by exhaustion. For each individual, the two trials were spaced 24 hr apart to allow each frog time to recover. Trial day and temperature trial order (i.e., whether the animal was in the constant or decreasing trial first) were randomly chosen across all individuals of both species. For each trial, a frog was placed in an open 250‐mL beaker with 10 ml of reconstituted RO water, allowing the frog to be partially submerged while resting on the floor of the beaker. The beaker was partially submerged in a recirculating water bath (Neslab RTE‐210, Thermo Fisher Scientific, USA) containing a 50:50 ethylene glycol:water mixture. Beaker temperature was recorded with a temperature probe partially submerged in the beaker water. Once the animal was in the beaker, it was gently moved onto its back with forceps every 2.5 min (i.e., every time the temperature decreased by 1°C in the decreasing temperature trial).

Starting at 15°C, and each subsequent temperature decrease (or every 2.5 min for the constant trials), we allowed each frog 10 s to right itself and recorded the righting response as successful or unsuccessful. Therefore, the maximum number of righting responses for each individual in a given trial was 15 (down to 1°C in the decreasing temperature trial). We calculated the CT_min_ as the first temperature (measured in the beaker) that individuals failed to right themselves in the decreasing temperature trial (Navas et al., 2007). We assumed the beaker water temperature was equivalent to the animals’ internal temperature since the small size of the animals (<1.5 g) enables rapid heat transfer between their body and the water (Navas et al., 2007). Also, we did not attempt to attach a temperature monitor to the frogs due to their small size, which may have affected their righting response abilities (Navas & Araujo, 2000). Several *H*. *versicolor* individuals did not attempt to right themselves in the beaker and exhibited a death feigning response (Banta & Carl, 1967), so were given two attempts to right themselves in a gloved hand, where they assumed death‐feigning less often. Several times we documented no attempt to right themselves in the beaker with the individual in a death‐feigning posture after 15 s, and immediate (<1 s) righting in‐hand. All *H*. *versicolor* were given the opportunity to right themselves in hand over the course of each trial to standardize methods within the species. The number of righting responses was recorded for both in beaker and in‐hand. Each trial ended when an individual was unable to right itself in the beaker or hand (when applicable).

### 2.4 *Bd* Infection diagnostics

To test each animal for *Bd* infection, one rayon‐tipped culture swab (MW 113; Medical Wire and Equipment Co Ltd, Corsham, England) was passed along three areas of each frog for a total of 40 swipes (10× on ventrum, 10× on each inner thigh, 1× under each toe) (Hyatt et al., 2007). Swabbing occurred immediately following their second righting response trial. The swab was placed in a 1.5‐ml microcentrifuge tube and stored in a −20°C freezer until DNA extraction. We did not swab frogs prior to righting response trials as to not disturb their thermal acclimation or increase stress responses, which can cause immunosuppression (Padgett & Glaser, 2003).

We extracted *Bd* DNA from swabs using PrepMan Ultra (Applied Biosystems by Life Technology Corporation, Carlsbad, CA) and quantified *Bd* using a quantitative polymerase chain reaction TaqMan assay (Boyle et al., 2004). Each sample was run in duplicate and considered positive for *Bd* if amplification occurred in both replicates on a StepOnePlus Real‐Time PCR system (Applied Biosystems, Foster City, CA). A sample was re‐run in duplicate if it tested positive in only one well, and subsequently classified as positive for *Bd* if amplification occurred in two of four wells. Infection load was quantified using gBlocks (Integrated DNA Technologies, Coralville, IA, USA) for *Bd* ITS genes as standards, which included four serial dilutions in duplicate in each plate (1,000 to 1×).

All methods were conducted under permission of the Purdue Animal Care and Use Committee (#1711001645) and the Indiana Department of Natural Resources Scientific Purposes License (#18‐099).

### 2.5 Statistical analysis

We first constructed a generalized linear mixed‐effects model (GLMM) with Poisson error distribution to test the effects of exposure status, species, and final mass, on the CT_min_ (analyzed as an integer as righting responses were tested after each 1°C decrease, e.g., 15°C, 14°C, 13°C). Individual frog ID and trial day were included as crossed random predictors. Including species as a factor in these models is solely to compare these two species and is not a comparison to indicate species diversity in CT_min_. All explanatory variables were tested for multicollinearity using variance inflation values (VIF), and a cutoff value of five was used to consider removing collinear variables (James et al., 2013). No explanatory variables were collinear, thus, all three remained in the GLMM. We constructed a second GLMM with Poisson error distribution to test the effects of species, temperature trial (constant or decreasing), and exposure status on the maximum number of times an individual was able to right itself in each trial. Exposure status and temperature trial were included as fixed predictors, and individual ID and trial day were included as crossed random predictors using the “lme4” package (Bates et al., 2015). Model assessment for each full model was ranked by Akaike's information criterion with a correction for finite sample size (AICc) with a threshold ΔAIC of two for distinguishing differences among models (Burnham & Anderson, 2002). For each response variable, a set of GLMMs starting with a global model that included all variables were created using the “lme4” package (Bates et al., 2015). We also compared differences in final mass and mass change between species, and between exposure statuses within species using Mann–Whitney U tests, as normalization via transformations was not achieved.

We also created a Cox proportional hazards model (Cox, 1972) to compare mortality in the *Bd*‐exposure period (before the righting response trials) between species, exposure status, and their interaction using the “survival” and “survminer” packages (Kassambara et al., 2019; Therneau, 2019). All analyses were conducted in R version 3.6.0 (R Core Team, 2018).

## 3 RESULTS

**FIGURE 1:**
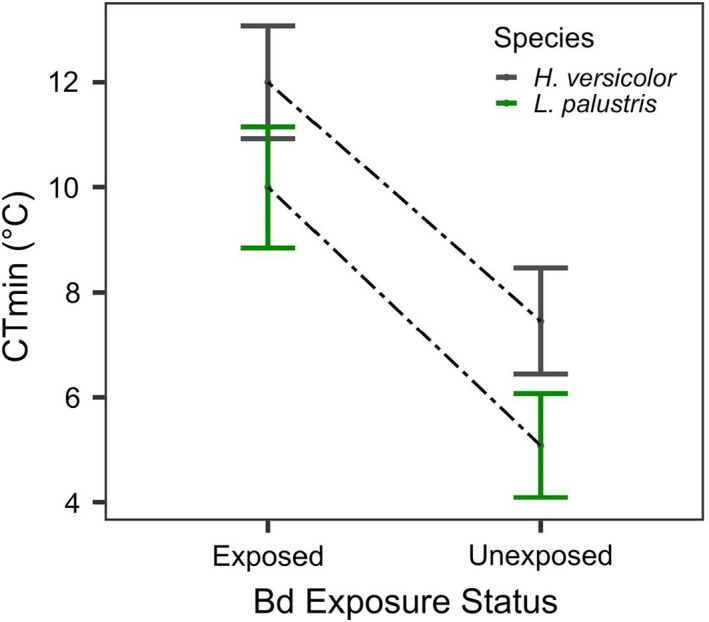
Comparison of exposure status and critical thermal minimum (CT_min_) between *Hyla versicolor* (gray) (*N* = 7 exposed, *N* = 11 unexposed), and *Lithobates palustris* (green) (*N* = 6 exposed, *N* = 12 unexposed). Individuals in the *Bd*‐exposed treatment had a significantly higher CT_min_ than individuals in the unexposed treatment. Additionally, *H*. *versicolor* had a higher CT_min_ than *L*. *palustris*

The best fit models predicting CT_min_ revealed that exposure status and species were significant predictors (Table 1). We found that *Bd* exposure increased the CT_min_ of both *H*. *versicolor* and *L*. *palustris*. In both species, unexposed individuals were able to right themselves at significantly lower temperatures than exposed individuals (*p* < .001, Table 1, Figure 1). Exposure to *Bd* resulted in a CT_min_ reduction of 60.86% (+4.54°C) for *H*. *versicolor*, and 96.9% (+4.92°C) for *L*. *palustris* (Figure 1). CT_min_ was higher for *H*. *versicolor* than *L*. *palustris* in both exposed and unexposed groups (*p* = .034, Table 1, Figure 1). Final mass did not influence CT_min_ in the final model; however, a Mann–Whitney U test revealed mean final mass of *Bd*‐exposed *L*. *palustris* (0.73 g [0.04 *SE*]) was significantly higher than *Bd*‐exposed *H*. *versicolor* (0.44 g [0.02 *SE*), (W = 6.42, *p* < .01). Final mass of unexposed *L*. *palustris* (0.83 g [0.04 *SE*]) was also significantly higher than unexposed *H*. *versicolor* (0.46 g [0.03 *SE*]) (W = 130, *p* < .001). We documented infection in three *H*. *versicolor* individuals from the *Bd*‐exposed treatments, with an average infection load of 4.74 genome equivalents/individual. Control individuals (unexposed *n* = 23) were also tested for infection and were all negative. Because only *H*. *versicolor* were infected (*n* = 3), we conducted a two‐sample *t*‐test to compare CT_min_ between infected and uninfected (of only exposed group) individuals and found no difference in mean CT_min_ between infected (12.7°C, [1.2 *SE*]) and uninfected (11.5°C, [0.91 *SE*]) individuals (*t*_(4.9)_ = −0.55, *p* = .61).

**TABLE 1:** Predictor variables from best supported generalized linear mixed‐effects model (GLMM) predicting critical thermal minimum (CT_min_). The top three GLMMs with AIC scores are listed. LRT = Likelihood ratio test of fixed predictors. *p*‐Values were derived from *drop1* function to test single fixed effects. Statistically significant explanatory variables are listed in bold

Models	*df* ^a^	AICc^b^	ΔAICc^c^
Exposure Status + Species	5	195.1	0.0
Exposure Status + Species + Final Mass	6	195.3	0.2
Exposure Status * Species	6	197.1	2.0
Exposure Status * Species * Final Mass	7	198.0	2.9
Exposure Status + Final Mass	5	198.8	3.8
Exposure Status * Final Mass	5	198.8	3.8
Species + Final Mass	5	202.9	7.8
Species * Final Mass	5	202.9	7.8

Explanatory variables	*df*	LRT^d^	*p*‐Value
1. Exposure Status	1	11.61	**<.001**
2. Species	1	8.02	**<.01**

^a^ *df* = Degrees of freedom

^b^ AIC = Akaike information criterion to rank candidate models, lower values denote more robust models.

^c^ ΔAIC = Delta Akaike information criterion, to measure relative differences between candidate models. Values ≥2 indicate candidate model is not as good as top model.

^d^ LRT = Likelihood ratio test of fixed predictors.

**FIGURE 2:**
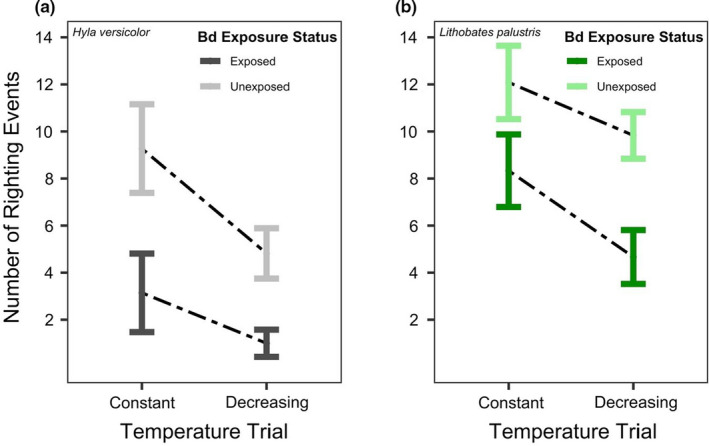
The number of righting events in each temperature trial for exposed and unexposed individuals for (a) *Hyla versicolor* (*N* = 7 exposed, *N* = 11 unexposed), and (b) *Lithobates palustris* (*N* = 6 exposed, *N* = 12 unexposed). Exposure to *Bd* and the decreasing temperature trial significantly reduced the number of righting events, but there were no differences between species

In both species, unexposed individuals righted themselves more times than exposed individuals across temperature trials (*p* < .001; compare exposed and unexposed treatments in Table 2, Figure 2a and b). Additionally, individuals righted themselves more times in the constant (15°C) trials compared to their decreasing trials (*p* < .001; compare temperature regimes in Figure 2a and b). Although the average number of righting events was lower across treatments and trials for *H*. *versicolo*r compared to *L*. *palustris*, there was no effect of species on the number of righting events in our model (*p* = .093, Table 2). There were no observations during trials for either species exhibiting muscular spasms, rigor, or death. Several frogs of both species exhibited poor posture when moved onto their back after instances of pulling their limbs tight to their body.

**TABLE 2:** Predictor variables from best supported generalized linear mixed‐effects model (GLMM) predicting number of times each individual could right itself. The top two GLMMs and interaction model of best GLMM with AIC scores are listed. LRT = Likelihood ratio test of fixed predictors. *p*‐Values were derived from *drop1* function to test single fixed effects. Statistically significant explanatory variables are highlighted in bold

Models	*df* ^a^	AIC^b^	ΔAIC^c^
Temperature Trial + Exposure Status + Species	6	416.9	0.0
Temperature Trial + Exposure Status	5	417.3	0.4
Temperature Trial * Exposure Status * Species	10	421.9	5.1

Explanatory Variables	*df*	LRT^d^	*p*‐Value
**1. Exposure Status**	1	11.40	**<.001**
**2. Temperature Trial**	1	21.42	**<.001**
3. Species	1	2.82	.093

^a^ *df* = Degrees of freedom

^b^ AIC = Akaike information criterion to rank candidate models, lower values denote more robust models.

^c^ ΔAIC = Delta Akaike information criterion, to measure relative differences between candidate models. Values ≥2 indicate candidate model is not as good as top model.

^d^ LRT = Likelihood ratio test of fixed predictors.

**FIGURE 3:**
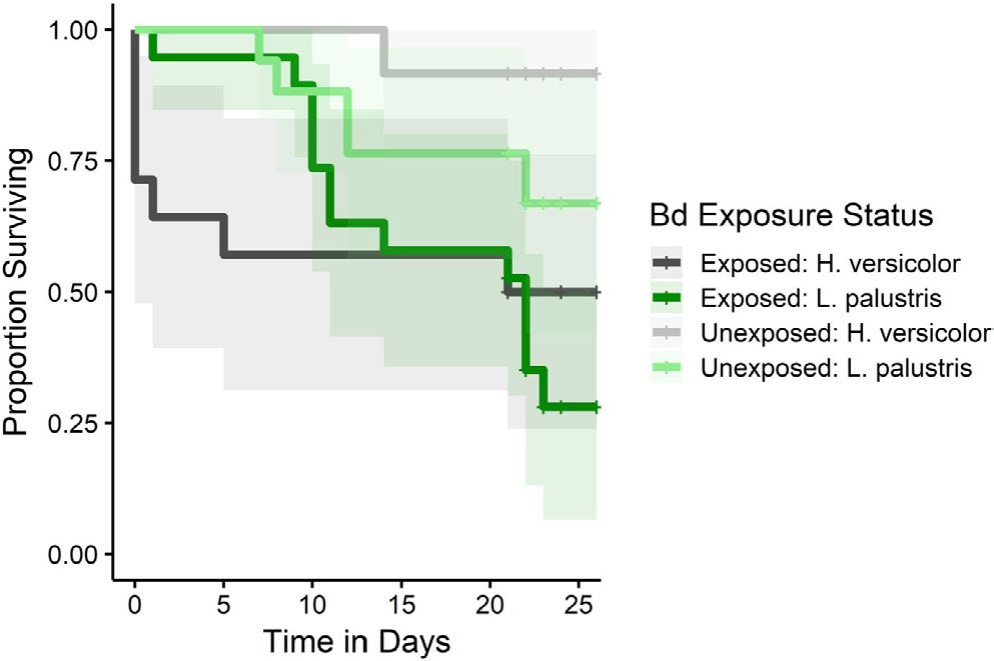
Survival in the pretrial periods across species and exposure status (±95% CI). *Bd* exposure reduced survival in both species, but there was no difference in survival between species

Exposed individuals had significantly lower survival in the pretrial period than unexposed individuals (*p* = .029), but there was no effect of species (*p* = .811) or the species by exposure treatment interaction (*p* = .249; Figure 3). In both species, the exposed group had an approximately 10 times greater chance of death than the unexposed group (hazard ratio = 10.3, *p* = .029).

Mann–Whitney *U* tests showed that, of the individuals that survived the entire experiment, final mass of *L*. *palustris* (0.73 g, 0.04 *SE*) was significantly greater than *H*. *versicolor* (0.44 g, 0.02 *SE*) in the *Bd*‐exposed groups (W = 42, *p* = .003). Final mass for unexposed *L*. *palustris* (0.83 g, 0.04 *SE*) was also significantly greater than *H*. *versicolor* (0.46 g, 0.03 *SE*; W = 130, *p* < .001). Further, *Bd*‐exposed *L*. *palustris* lost significantly more mass (−24.6%) than unexposed *L*. *palustris* (−17.3%) throughout the study (W = 11, *p* = .02), but there were no differences in mass change between exposed and unexposed *H*. *versicolor*. Starting mass and final mass did not differ between exposed and unexposed individuals within either species.

## 4 DISCUSSION

We found that pathogen exposure increased the CT_min_ of both amphibian species and led to fewer righting responses in both the decreasing and constant temperature trials. Because individuals were able to right themselves more times in the constant than decreasing trials, our results indicate that the higher CT_min_ of exposed individuals (Figure 1) was due to the colder temperatures of the decreasing trial and not exhaustion (i.e., they had the potential for more righting events if the temperature was not decreasing). Overall, our results show that pathogen exposure can affect righting response and increase CT_min_, which may reduce an individual's ability to function and survive in winter conditions.

We found an effect of *Bd* exposure on CT_min_ and the number of successful righting responses in both species despite low detection of *Bd* on exposed frogs when the trials concluded. Of the 36 exposed individuals, we only found *Bd* infections on three *H*. *versicolor* with an average infection load of 4.74 genome equivalents. Although the positive infections indicate the Bd was viable, it is possible exposures were not successful at infecting the frogs. However, Bd exposure has been shown to induce disease‐related effects (e.g., tissue degradation) in the absence of infection through zoospore secretions of proteins (Brutyn et al., 2012; McMahon et al., 2013; Moss et al., 2010). Alternatively, the paucity of infection by the time of the trials may have been caused by the exposed frogs resisting (or clearing) infection during the 21‐day pretrial period. The frogs in our experiment could have resisted infections through innate immune responses, such as anti‐*Bd* bacteria that compete with *Bd* or produce growth‐inhibiting properties, or antimicrobial peptides (AMPs) in skin secretions that inhibit *Bd* growth (Grogan, Robert, et al., 2018; Pask et al., 2013; Rollins‐Smith & Conlon, 2005). However, anti‐*Bd* bacteria activity is reduced in lower temperatures (down to 8°C) in vitro (Daskin et al., 2014), suggesting resistance via bacteria is limited during cold periods (i.e., winter). Amphibians can also mount adaptive immune responses to *Bd* via cell mediated, and humoral immunity, that lead to pathogen resistance (Grogan, Robert, et al., 2018; Rollins‐Smith et al., 2009). Therefore, there are multiple mechanisms that could have allowed the frogs in our study to resist or clear *Bd* infection.

The increased CT_min_ of *Bd*‐exposed individuals suggests that pathogen exposure impairs host behavioral response in cooling conditions. Our metric of a righting response to measure behavioral responsiveness requires neuromuscular coordination, which can be reduced in a frog species due to cooling temperatures (Costanzo et al., 1991) and *Bd* infection (Berger et al., 2005). For frogs infected with *Bd*, Andre et al. (2008) reported unresponsiveness was more common at cooler temperatures (17°C vs. 22°C), likely because the hosts were better able to immunologically cope with infection at warmer temperatures (i.e., resistance), while *Bd* growth and reproduction were unchanged between the temperatures in this study. We show that when combined, cooling temperatures and *Bd* exposure can have similar effects, suggesting temperature and *Bd* act synergistically to impair frogs’ behavioral responsiveness. Our results can only imply behavioral responsiveness was affected, as opposed to physiological collapse (e.g., muscular spasms) and we did not observe signs of physiological collapse in any individuals, likely because of limited and low‐level infections (Greenspan et al., 2017). Additionally, physiological collapse is not a common or recommended measure of CT_min_ (Lutterschmidt & Hutchison, 1997; Taylor et al., 2020). However, several frogs of both species were unable to pull their limbs into their body when moved onto their back after being able to do so earlier in the trial. Other factors such as impaired force development due to reduced muscle and fiber growth (Fitts et al., 1991) and the sympathetic nervous system may also impact behavioral responsiveness, particularly in developing juvenile frogs.

Individuals exposed to a pathogen may undergo resource trade‐offs between immune responses and physiological maintenance to tolerate cold conditions (Barribeau et al., 2008). The energetic stores needed for an immune response to *Bd* may have induced physiological and survival costs, possibly explaining the higher CT_min_ and mortality we observed in *Bd*‐exposed frogs (Bonneaud et al., 2003) (Figures 1 and 3). Competition of energetic stores for immune responses, as well as growth and activity, likely worsens when amphibians enter overwintering acclimation. Amphibians reduce physiological and immunological functions, while also decreasing food intake to replenish energy expenditures (Beck & Congdon, 2000; Resetarits, 1986). The reduction in physiological and immunological performance of *H*. *versicolor* and *L*. *palustris* occurs well above the temperatures at which *Bd* slows growth and reproduction (~4°C), suggesting that hosts must elicit an immune response under suboptimal physical conditions (John‐Adler et al., 1988; Resetarits, 1986; Voyles et al., 2017). Therefore, immunocompromised individuals may be expending greater energetic stores to combat *Bd* due to cold temperatures, leading to reduced cold tolerance and higher mortality. The scarcity of resources available to combat infection or maintain metabolic function in winter could be driving unobserved *Bd*‐related mortality.

Our finding that *Bd* exposure impaired locomotor function at cold temperatures could affect life history behaviors of *H*. *versicolor* and *L*. *palustris* that ultimately induce physiological and fitness costs. It is reported that *L*. *palustris* often overwinter in caves and remain active at temperatures around ≤6°C (Resetarits, 1986), similar to the 5.1°C CT_min_ for unexposed individuals in our study. However, the loss of locomotor function we documented at 10°C for exposed *L*. *palustris* (Figure 1) suggests *Bd* exposure could reduce activity sooner, resulting in possible freezing if individuals are unable to reach overwintering sites, or could lead to starvation if they are unable to capture prey (Resetarits, 1986). For *H*. *versicolor*, this species will move between arboreal refugia and the forest floor (overwintering microhabitats) as temperatures drop and fluctuate in the fall (Ritke & Babb, 1991; Roble, 1979; Storey & Storey, 1985), and they must produce enough cryoprotectant enzymes to tolerate freezing the majority of their body (Storey & Storey, 1985). Therefore, we speculate that a reduction in physiological abilities after *Bd* exposure may impair movement between microhabitats, and the capacity to feed and produce cryoprotectants (Sibly & Calow, 1986; Sinclair et al., 2013), potentially resulting in mortality due to starvation or inability to tolerate freezing.

Beyond localized movement patterns, an elevated behavioral CT_min_ could signify a compressed species distributional limit, spatially and temporally. Populations of both *H*. *versicolor* and *L*. *palustris* reach into south eastern Canada (Dodd, 2013), where temperatures fall below 10°C for 7 months (en.climate‐data.org), and these regions have documented *Bd* infections (Ouellet et al., 2005). If CT_min_ increases for both species, northern range limits may be compressed. Additionally, northern populations that must endure colder temperatures may be at greater risk of range compression, and mortality related to *Bd* exposure. *H*. *versicolor* reaches higher latitudes, but is more sensitive to cold when exposed to *Bd*, and therefore may experience a greater northern range reduction than *L*. *palustris*. However, testing thermal limits of local population in northern latitudes is necessary to better predict potential distributional changes due to *Bd*.

The use of juveniles in our study could have specific age‐class responses to *Bd* exposure and cold tolerance. The CT_min_ of 8.3°C (unexposed) and 12°C (exposed) for *H*. *versicolor* appears high relative to some measures of the activity in natural populations (John‐Adler et al., 1988). However, juvenile *H*. *versicolor* produce lower concentrations of cryoprotectants than adults, suggesting the immature age class is predisposed to have a lower cold tolerance than adults (Storey & Storey, 1985). Likely due to smaller size, juvenile *L*. *palustris* in natural populations are at greater risk of mortality than adults, causing younger individuals to be less capable of surviving winter (Resetarits, 1986).

The size differences between the *H*. *versicolor* and *L*. *palustris* in our study could be driving the species effect in CT_min_. Larger mass has been shown to decrease *Bd*‐induced mortality in metamorphs (*H*. *versicolor* and *L*. *pipiens*; Searle et al., 2011), and lower the probability of being infected with *Bd* (Murray et al., 2013). Since *L*. *palustris* is a larger species, these individuals might more successfully handle infections, explaining our result that *L*. *palustris* righted themselves more and retained a lower CT_min_ than *H*. *versicolor*. However, because only a single clutch was used per species, the differences found between species could be due to a clutch (i.e., family) effect and not species. Therefore, a more robust interpretation of species would be possible by comparing multiple unrelated clutches.

## 5 CONCLUSION

Our results showed that pathogen exposure can increase the CT_min_ of hosts, which may reduce their ability to survive and function in winter conditions. During the winter, the extent to which cold temperatures lead to immunosuppression or energetic trade‐offs between immune responses and physiological maintenance must be considered in future investigations to fully understand disease risk. Susceptibility to pathogen‐related effects is highly context dependent, varying across host species, age, sex, family, and spatial distributions. Therefore, studies must examine a diverse array of hosts, potentially those that exhibit characteristics that make them most susceptible to disease, such as juveniles with underdeveloped immune systems. Drivers and consequences of disease dynamics in winter often go unnoticed or untested, especially for species that overwinter in cryptic microhabitats. However, our study highlights the need to employ greater effort to monitor the effects of pathogens on winter performance and survival, which has been largely understudied.

## CONFLICT OF INTEREST

No competing interests declared.

## AUTHOR CONTRIBUTIONS

**Spencer R. Siddons:** Conceptualization (equal); Data curation (lead); Formal analysis (lead); Funding acquisition (equal); Investigation (lead); Methodology (equal); Project administration (lead); Resources (equal); Software (lead); Supervision (equal); Validation (equal); Visualization (lead); Writing‐original draft (lead); Writing‐review & editing (lead). **Catherine L. Searle:** Conceptualization (equal); Data curation (supporting); Formal analysis (supporting); Funding acquisition (equal); Investigation (supporting); Methodology (equal); Project administration (supporting); Resources (equal); Software (supporting); Supervision (lead); Validation (equal); Visualization (supporting); Writing‐original draft (supporting); Writing‐review & editing (supporting).

## Data Availability

Data are available on Dryad Digital Repository, https://doi.org/10.5061/dryad.sqv9s4n43.
